# Dysfunctional ADAM22 implicated in progressive encephalopathy with cortical atrophy and epilepsy

**DOI:** 10.1212/NXG.0000000000000046

**Published:** 2016-01-21

**Authors:** Mikko Muona, Yuko Fukata, Anna-Kaisa Anttonen, Anni Laari, Aarno Palotie, Helena Pihko, Tuula Lönnqvist, Leena Valanne, Mirja Somer, Masaki Fukata, Anna-Elina Lehesjoki

**Affiliations:** From the Institute for Molecular Medicine Finland (M.M., A.P.), Neuroscience Center (M.M., A.L., A.-E.L.), and Research Programs Unit, Molecular Neurology (M.M., A.-K.A., A.L., A.-E.L.), University of Helsinki, Finland; Folkhälsan Institute of Genetics (M.M., A.-K.A., A.L., A.-E.L.), Helsinki, Finland; Division of Membrane Physiology (Y.F., M.F.), Department of Cell Physiology, National Institute for Physiological Sciences, National Institutes of Natural Sciences, Okazaki, Japan; Department of Physiological Sciences (Y.F., M.F.), School of Life Science, SOKENDAI (The Graduate University for Advanced Studies), Okazaki, Japan; Medical and Clinical Genetics (A.-K.A.), University of Helsinki and Helsinki University Hospital, Helsinki, Finland; Analytic and Translational Genetics Unit (A.P.), Department of Medicine, Massachusetts General Hospital and Harvard Medical School, Boston, MA; Program in Medical and Population Genetics (A.P.) and Stanley Center for Psychiatric Research (A.P.), Broad Institute of Harvard and Massachusetts Institute of Technology, Cambridge, MA; Program in Genetics and Genomics (A.P.), Biological and Biomedical Sciences, Harvard Medical School, Boston, MA; Wellcome Trust Sanger Institute (A.P.), Wellcome Trust Genome Campus, Hinxton, United Kingdom; Psychiatric & Neurodevelopmental Genetics Unit (A.P.), Department of Psychiatry, and Department of Neurology (A.P.), Massachusetts General Hospital, Boston, MA; Department of Pediatric Neurology (H.P., T.L.), Children's Hospital, University of Helsinki and Helsinki University Hospital, Helsinki, Finland; Department of Radiology (L.V.), HUS Medical Imaging Center, Helsinki, Finland; and Family Federation of Finland (M.S.), Helsinki, Finland.

## Abstract

**Objective:**

To identify the molecular genetic basis of a syndrome characterized by rapidly progressing cerebral atrophy, intractable seizures, and intellectual disability.

**Methods:**

We performed exome sequencing in the proband and whole-genome single nucleotide polymorphism genotyping (copy number variant analysis) in the proband-parent trio. We used heterologous expression systems to study the functional consequences of identified mutations.

**Results:**

The search for potentially deleterious recessive or de novo variants yielded compound heterozygous missense (c.1202G>A, p.Cys401Tyr) and frameshift deletion (c.2396delG, p.Ser799IlefsTer96) mutations in *ADAM22*, which encodes a postsynaptic receptor for LGI1. The deleterious effect of the mutations was observed in cell surface binding and immunoprecipitation assays, which revealed that both mutant proteins failed to bind to LGI1. Furthermore, immunoprecipitation assays showed that the frameshift mutant ADAM22 also did not bind to the postsynaptic scaffolding protein PSD-95.

**Conclusions:**

The mutations identified abolish the LGI1-ADAM22 ligand-receptor complex and are thus a likely primary cause of the proband's epilepsy syndrome, which is characterized by unusually rapidly progressing cortical atrophy starting at 3–4 months of age. These findings are in line with the implicated role of the LGI1-ADAM22 complex as a key player in nervous system development, specifically in functional maturation of postnatal synapses. Because the frameshift mutation affects an alternatively spliced exon with highest expression in postnatal brain, the combined effect of the mutations is likely to be hypomorphic rather than complete loss of function. This is compatible with the longer survival of the patient compared to *Lgi1*^−/−^ and *Adam22*^−/−^ mice, which develop lethal seizures during the first postnatal weeks.

Many types of epilepsies are caused by genetic defects in ion channel or neurotransmitter genes, but an alternative epileptogenic mechanism is revealed by dysfunction of the complexes formed by the secreted neuronal glycoprotein LGI1 and its pre- and postsynaptic receptors ADAM23 and ADAM22, respectively.^1,2^ ADAM22 and ADAM23 are members of the ADAM (A Disintegrin And Metalloproteinase) family of transmembrane proteins implicated in proteolysis and cell adhesion.^3^ Unlike many ADAM proteins, neuronally expressed ADAM22 and ADAM23 are catalytically inactive and act by binding to other proteins. LGI1-ADAM22 and LGI1-ADAM23 complexes have recently been suggested to function as transsynaptic players forming macromolecular complexes regulating synapse maturation and function, particularly in postnatal brain^1,2,4,5^ (reviewed in reference 6). In addition to the postsynaptic membrane, ADAM22 also functions in axons in the CNS^7^ and peripheral nervous system (PNS)^8^ and in Schwann cells.^8^

LGI1, ADAM22, and ADAM23 are all genetically linked to epilepsy. Heterozygous mutations in *LGI1*, which impair LGI1 secretion or binding to its receptors,^9^ cause autosomal dominant lateral temporal lobe epilepsy (ADLTE; OMIM #600512).^10^ Although *ADAM22* and *ADAM23* mutations have not been reported in human diseases, knockout mice for *Lgi1*,^2,11,12^ *Adam22*,^13^ and *Adam23*^14^ manifest with lethal seizures during the first postnatal weeks. Furthermore, *ADAM23* has been implicated in canine epilepsy.^15^

Advances in high-throughput sequencing methods have enhanced identification of genes for rare disorders, including epilepsy syndromes.^16–18^ We used whole-exome sequencing to characterize the genetic cause of a severe infantile-onset progressive encephalopathy with intractable seizures. We discovered compound heterozygous mutations in *ADAM22* that compromise the protein function.

## METHODS

### Study patient.

The proband was ascertained from a series of 30 Finnish patients with severe epilepsy syndromes who underwent whole-exome sequencing. The clinical data were reviewed from hospital records. H.P. and M.S. personally examined the patient. The original CT and MRI images obtained prior to the age of 11 years were not available for re-review.

### Standard protocol approvals, registrations, and patient consents.

An institutional review board at the Helsinki University Hospital approved the study. Informed consent for DNA analysis was obtained from the parents.

### Exome sequencing and variant calling.

Genomic DNA extracted from peripheral blood of the proband was exome-sequenced at the Wellcome Trust Sanger Institute (Hinxton, Cambridge, United Kingdom) using methods described previously.^19^ Briefly, SureSelect Human All Exon 50 Mb V3 RNA baits (Agilent Technologies, Santa Clara, CA) were used to enrich exonic targets, and sequencing was carried out using HiSeq 2000 platform (Illumina, San Diego, CA). Alignment of the sequence reads to Human Reference Genome hs37d5 (based on GRCh37) and their further processing were performed as described previously.^19^ Single nucleotide variants and indels of the proband exome were called jointly with 84 previously published exomes^19^ and 42 unpublished in-house exomes using GATK HaplotypeCaller (v. 3.3; https://www.broadinstitute.org/gatk/).^20–22^

### Variant analysis under recessive and de novo inheritance models.

Reflecting the different possible inheritance patterns of the underlying mutation(s) in the study patient with no affected relatives, rare autosomal or sex-linked recessive and novel heterozygous de novo mutations were explored. Mutations in mitochondrial genome were also analyzed. The variant filtering process is described in detail in appendix e-1 and figure e-1 at Neurology.org/ng.

### Variant validation and segregation analysis.

Candidate variants were confirmed, and segregation of the variants was analyzed by bidirectional Sanger sequencing. Primers are available from the authors upon request.

### Analysis of copy number variants.

Illumina Human CoreExome single nucleotide polymorphism array with 548K markers enriched to genic regions was used to detect copy number variants (CNVs) from genomic DNA isolated from peripheral blood of the proband and parents and to confirm correct relatedness of DNA samples in the proband-parent trio. PennCNV^23^ was used to call CNVs (http://www.openbioinformatics.org/penncnv/).

### Functional studies.

#### Plasmid construction.

The complementary DNA (cDNA) of human *LGI1* was purchased from Thermo Scientific (clone ID: 4811956) and subcloned into pCAGGS with FLAG tag at its C-terminus. The cDNA of human *ADAM22* (same sequence as in NM_021723 except the c.242C>G, p.Pro81Arg polymorphism in the Pro domain, which is cleaved from the mature ADAM22) was subcloned into pCAGGS. ADAM22 mutants Cys401Tyr and Ser799IlefsTer96 were generated by using site-directed mutagenesis. pGW1-PSD-95-FLAG was constructed by replacing a green fluorescent protein (GFP) fragment of pGW1-PSD-95-GFP with a synthetic DNA fragment encoding FLAG.^24^ All PCR products were verified by DNA sequencing.

#### Antibodies.

The following antibodies were used: rabbit polyclonal antibodies to LGI1 (ab30868; Abcam, San Francisco, CA), mouse monoclonal antibodies to KDEL (10C3; Enzo Life Sciences, New York, NY), PSD-95 (MA1-046; Thermo Scientific, Waltham, MA), and FLAG (M2; Sigma-Aldrich, St. Louis, MO). Rabbit polyclonal antibodies to ADAM22 were raised against GST-mouse ADAM22 (aa 444-526), corresponding to the extracellular disintegrin domain. Antibodies were affinity-purified on a cyanogen bromide–activated Sepharose 4B (GE Healthcare, Piscataway, NJ) column containing the immunizing antigen.

#### Cell surface binding assay.

The assay was performed in COS7 cells with minor modifications to a previously reported method.^1^ Details of the assay are presented in appendix e-1.

#### Immunoprecipitation.

HEK293T cells were cotransfected with wild-type LGI1-FLAG or PSD-95-FLAG together with either wild-type ADAM22 or one of the ADAM22 mutants, Cys401Tyr or Ser799IlefsTer96. The immune complexes were precipitated with FLAG-M2 agarose and separated by sodium dodecyl sulfate polyacrylamide gel electrophoresis. Gels were subjected to Western blotting. A more detailed description of the method is presented in appendix e-1.

### Accession codes.

The raw aligned sequence reads were submitted to the European Genome-phenome Archive (https://www.ebi.ac.uk/ega/home) by Wellcome Trust Sanger Institute under study accession ID EGAS00001000190.

## RESULTS

### Clinical features.

The patient, currently 26 years of age, was born at term after an uncomplicated pregnancy. She is the second child of healthy nonconsanguineous parents of Finnish ancestry, with no family history of encephalopathy or epilepsy. During pregnancy, fetal movements were normal. Birth measurements were 3,700 g/51 cm (+0.5 SD)/33.5 cm (−1 SD). She had an Apgar score of 10 and was discharged from the hospital normally. The first signs of disease were brief episodes of apnea at 2 months of age, and soon thereafter she lost her ability to smile and make eye contact. At 3 months, she was hospitalized for repeated brief seizures. The seizures started with focal symptoms and later generalized. EEG was severely abnormal, and generalized spikes initiating from the left side with abnormal background activity were seen. The seizures proved to be intractable; treatment with barbiturate anesthesia and phenobarbital, phenytoin, carbamazepine, and valproic acid in different combinations was ineffective. The ammonium levels were mildly and briefly elevated during hospitalization, but metabolic screening tests remained normal. At 3.5 and 4 months, the brain CT was normal, but 6 weeks later, marked supratentorial atrophy was present. From 6 to 9 months the seizures were better controlled, but thereafter she started to have short focal clonic seizures or myoclonias.

At 1 year of age, she was very hypotonic, with no active movement, and had brisk reflexes in the lower extremities and rigidity in both the upper and lower extremities. She continued to have short tonic seizures, sometimes with focal clonic jerks in her left eye and in the corner of the mouth. The occipital frontal circumference (OFC) was 43 cm (−2.5 SD). She had a narrow forehead, open mouth, full lips, and a high and narrow palate. The nose was short and the ear lobules were outward-turning, and she had intermittent swelling of extremities and face. Electroretinography was normal. On brain MRI, the supratentorial atrophy had progressed, and there was marked subdural effusion. Cerebellum and brain stem were normal. In morphologic assessment of muscle biopsy, nicotinamide adenine dinucleotide and cytochrome *c* stainings were normal, but frequent lipid vacuoles were seen. Electron microscopic analysis of the sural nerve was normal, and electroneuromyography also showed normal results. The liver enzymes were moderately elevated, serum lactate was slightly above normal range, and respiratory chain analysis revealed unspecific changes (low consumption of oxygen and high complex IV activity with cytochrome *c*) not exclusively pointing toward mitochondrial defect. Neuropathy, ataxia, retinitis pigmentosa syndrome point mutation analysis and mitochondrial DNA Southern blotting gave normal results.

**Figure 1:**
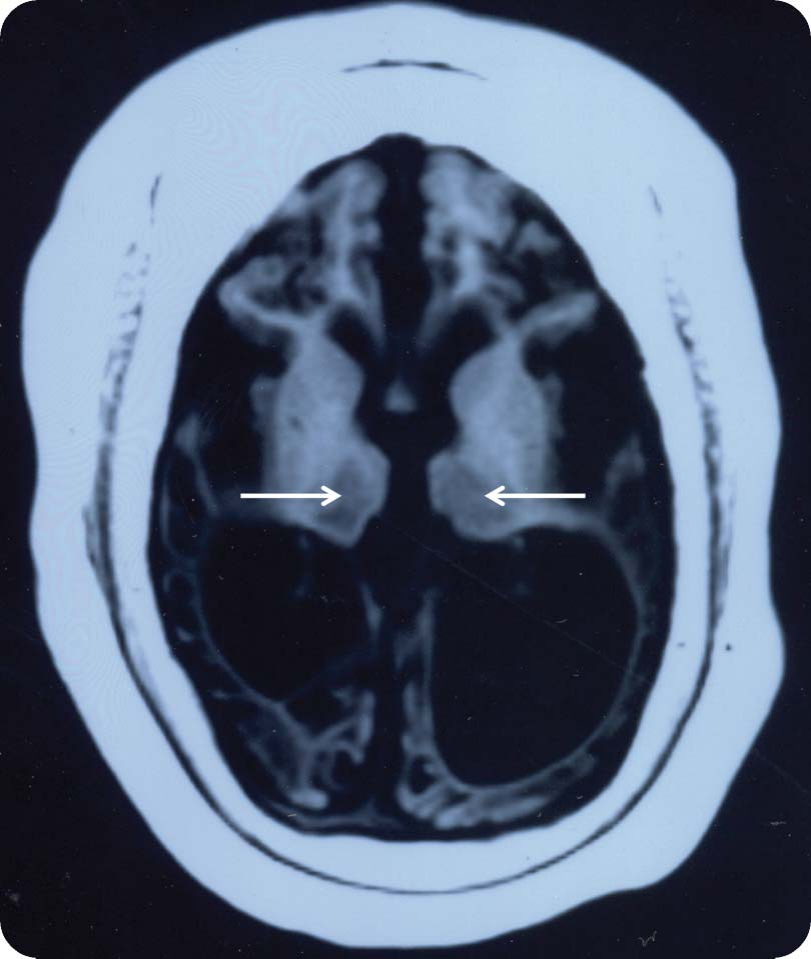
Brain imaging findings in the patient An axial T1-weighted MRI image of the patient at the age of 11 years. There is extensive loss of both gray and white matter with corresponding widening of the CSF spaces and thickening of the skull. Both thalami (arrows) show shrinking and signal change.

At 2 years, the OFC was 44.5 cm (−3 SD) and the CT showed marked supratentorial atrophy and subdural effusion. The cerebellum was normal. She experienced brief generalized seizures up to 4 times per day and was treated with phenobarbital, valproic acid, and diazepam. No voluntary movements were seen, and microcephaly together with profound intellectual disability was present. The ophthalmologic examination was normal at 2 years and showed mild atrophy of the optic nerve at 4 years 10 months. No signs of hearing loss were noted, and sometimes an auditory stimulus preceded a seizure. After the rapid progression during the first year of life, her epilepsy has remained stable with focal spikes and decreased background activity. The MRI images at 11 years showed severe cortical atrophy, widening of both central and cortical CSF spaces, and skull thickening (figure 1). She is currently nonambulatory, has intractable epilepsy, and is living in institutional care.

### Genetic analysis reveals compound heterozygous mutations in *ADAM22*.

**Figure 2:**
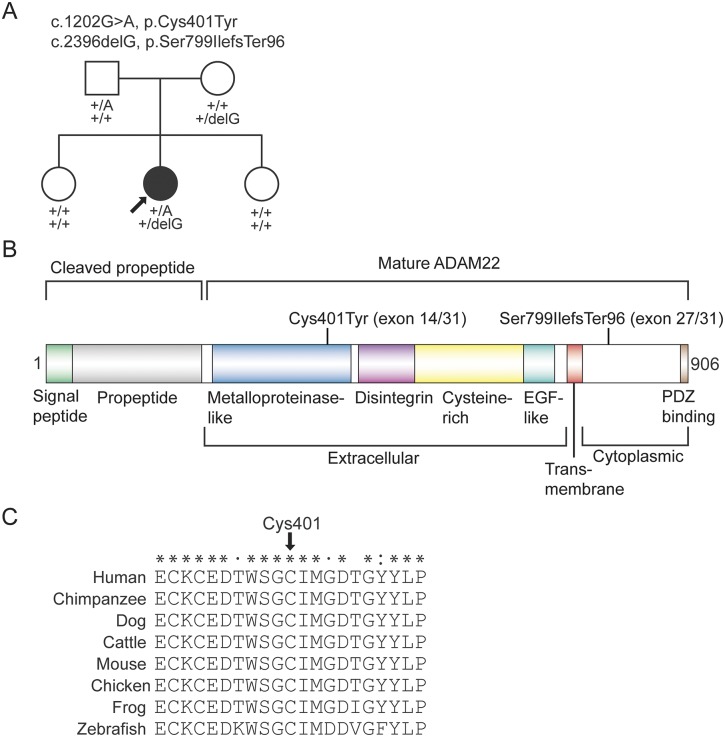
Compound heterozygous mutations in *ADAM22* (A) Segregation of the mutations in the family. Arrow indicates the exome-sequenced patient. +, wild-type. (B) Domain structure of ADAM22 and location of the mutations. Exon numbering and mutation positions are based on the longest isoform of *ADAM22* (variant 1, NM_021723). (C) ClustalX (http://www.clustal.org/clustal2/) comparison of amino acid sequences surrounding Cys401 shows full conservation of Cys401 (arrow) across different vertebrate species. Fully conserved, strongly similar, and weakly similar amino acid residues are labeled with asterisks, colon, and periods, respectively.

We analyzed the exome variant data of the proband (sequencing metrics in appendix e-1) for rare and potentially deleterious autosomal or X-linked recessive mutations (figure e-1). Variants in 2 genes, *ADAM22* and *CPA4*, passed the recessive filtering (figure e-1, table e-1), but only the mutations in *ADAM22* segregated in an autosomal recessive manner in the family (figure 2A and figure e-2). The patient was compound heterozygous for a missense mutation c.1202G>A encoding the p.Cys401Tyr substitution (mutation nomenclature based on *ADAM22* variant 1, RefSeq ID NM_021723) and a 1-bp frameshift deletion c.2396delG (p.Ser799IlefsTer96). The missense mutation is present in the heterozygous state in 2 individuals of European ancestry in the Exome Aggregation Consortium (ExAC) database (http://exac.broadinstitute.org/), yielding an allele frequency of 1.658 × 10^−5^. The deletion mutation is novel. The missense substitution, p.Cys401Tyr, occurs in a highly conserved cysteine residue that forms a disulfide bond with Cys394 in the metalloproteinase domain^25^ (figure 2, B and C and figure e-3). The p.Cys401Tyr substitution is predicted to be deleterious by all 4 in silico methods used. The frameshift deletion is predicted to alter the last ∼100 residues of ADAM22, thus abolishing the PDZ-binding motif (figure 2B). The deletion occurs in exon 27 of the longest isoform of *ADAM22* (RefSeq ID NM_021723), which encodes residues in the cytoplasmic tail and is subjected to alternative splicing^26,27^ (figures e-4 and e-5, see also Discussion).

Eight variants passed the filtering under the “de novo” hypothesis, where we looked for novel heterozygous variants (figure e-1, table e-1). Because the parents were not exome- sequenced, we analyzed segregation of these variants by capillary sequencing. All 8 variants were inherited from an unaffected parent and thus were not considered pathogenic. Variants in mitochondrial DNA were known polymorphisms (data not shown).

Analysis of CNVs in the proband-parent trio did not yield likely pathogenic variants (data not shown).

### ADAM22 mutants show aberrant binding to LGI1.

**Figure 3:**
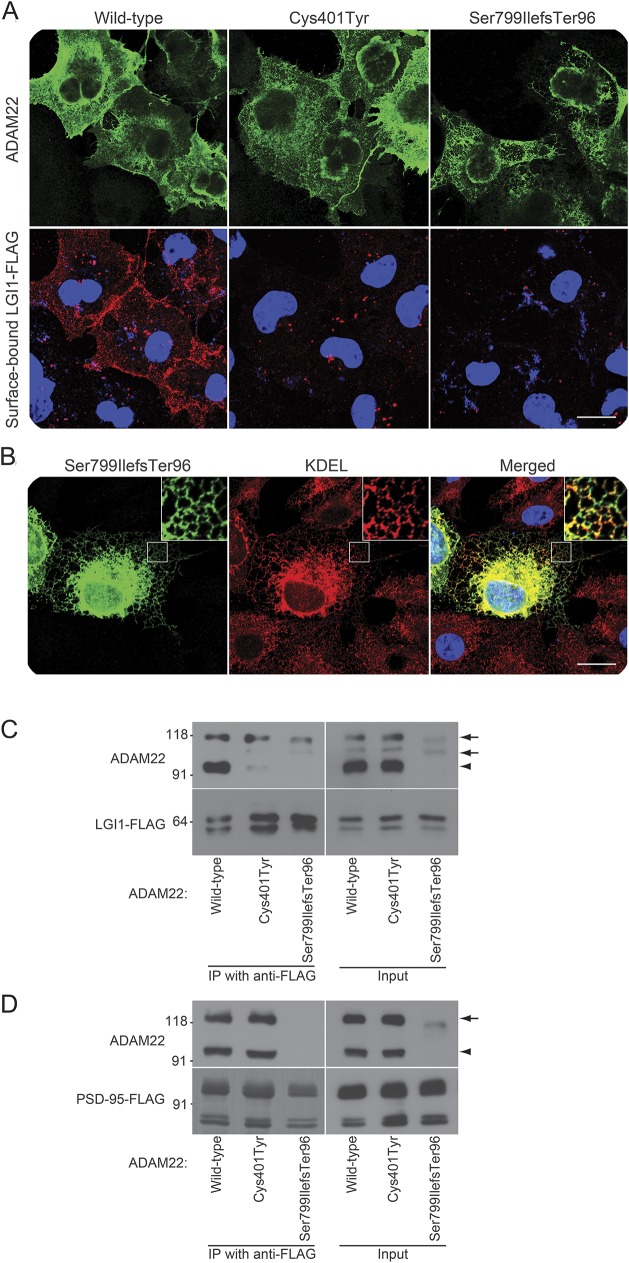
ADAM22 mutant proteins do not bind to LGI1 (A) Fluorescent confocal microscope images from the cell surface binding assay, in which indicated *ADAM22* complementary DNAs were cotransfected with wild-type FLAG-tagged *LGI1* into COS7 cells. Surface-bound FLAG-tagged LGI1 was labeled before cell permeabilization (red) and then ADAM22 was stained (green). Both ADAM22 mutants fail to bind to LGI1. Bar: 20 μm. (B) Ser799IlefsTer96 mutant (green) is localized in the endoplasmic reticulum labeled by the anti-KDEL antibody (red). Regions outlined with white squares are magnified in the upper right of the images. Bar: 20 μm. (C, D) Immunoprecipitation of ADAM22 mutants with FLAG-tagged LGI1 (C) or PSD-95 (D) in HEK293T cells. Neither ADAM22 mutant binds to LGI1 (left panel in C). The Cys401Tyr mutant binds to PSD-95, but the Ser799IlefsTer96 mutant does not (left panel in D). Arrows and arrowheads indicate the positions of immature and mature ADAM22, respectively. Immature ADAM22 is often observed in overexpressed cells and seems to nonspecifically bind to LGI1 under these conditions, whereas in the brain the immature band is not observed.

Using a cell surface binding assay, we first examined whether LGI1 interacts with the ADAM22 mutants. When LGI1 was cotransfected with wild-type ADAM22, LGI1 efficiently bound to ADAM22 on the cell surface as previously reported^1^ (figure 3A). In contrast, LGI1 did not bind to the Cys401Tyr and Ser799IlefsTer96 mutants on the cell surface. Although the localization of the Cys401Tyr mutant was similar to that of the wild type, most of the Ser799IlefsTer96 mutant protein was localized in the endoplasmic reticulum (ER), labeled by anti-KDEL antibody (figure 3, A and B).

We next analyzed the heterologous expression of *ADAM22* mutants in HEK293T cells by Western blotting. The expression level of Cys401Tyr was comparable to that of the wild type, whereas Ser799IlefsTer96 was less expressed in HEK293T cells and remained as an immature form (propeptide not cleaved, see inputs in figure 3, C and D). When LGI1 was cotransfected with wild-type ADAM22, ADAM22 was efficiently coimmunoprecipitated with LGI1 (figure 3C). In contrast, neither the Cys401Tyr nor the Ser799IlefsTer96 mutant was coimmunoprecipitated with LGI1 (figure 3C, arrowheads). We also found that the wild-type ADAM22 and Cys401Tyr mutant, which have a PDZ-binding motif at their C-terminus, bound to the postsynaptic scaffolding protein PSD-95, as shown previously for wild-type ADAM22,^1^ but Ser799IlefsTer96 lacking the motif did not (figure 3D).

## DISCUSSION

We identified compound heterozygous loss-of-function mutations in *ADAM22* in a patient with rapidly progressing severe encephalopathy with intractable seizures and profound intellectual disability. After initially normal CT scans, a remarkable feature of her disease was the rapidly progressing cerebral atrophy (within only a few weeks) with subdural effusions that became apparent approximately 2 months after seizure onset. The brain imaging changes were very similar to those seen in Menkes syndrome,^28,29^ and the patient's symptoms also resemble those seen in Alpers syndrome.^30^ The disease course of the patient was also unusual. The progression was very rapid in infancy after the onset of intractable seizures at 3 months of age, following the apparently normal first months of life. Thereafter the condition stabilized, leaving the patient nonambulatory with intractable seizures, and she is still alive at the age of 26 years. A rapidly progressing disease course in infancy may be a characteristic feature for *ADAM22*-associated disease, related to the role of ADAM22 in postnatal brain development.

We searched for pathogenic recessive and de novo variants in the exome and CNV data. The only variants passing the filtering and having a compatible segregation pattern in the family were compound heterozygous mutations in *ADAM22* (figure e-1). The ability to dramatically reduce the number of candidate variants when only the proband was exome-sequenced was possible by filtering against comprehensive variant databases including population-matched individuals, in our case Finns in the ExAC database.

Our functional assays indicated that both mutations cause a nonfunctional ADAM22 protein. The Cys401Tyr mutant did not bind to LGI1 in cell surface binding and immunoprecipitation assays. The p.Cys401Tyr substitution is predicted to break a disulfide bond in the metalloproteinase-like (M) domain. The crystal structure of the extracellular domain of ADAM22 has been recently determined^25^ (figure e-3). Based on this structure, the authors suggested a modular movement model of ligand binding to ADAM22, where the ligand, LGI1, competes with the M domain in binding with the cysteine-rich domain (C), making the M domain move away from the C domain.^25^ Because the p.Cys401Tyr substitution likely affects the tertiary structure of the M domain, it may hinder the conformational change that is necessary for ADAM22 in implementing its protein–protein adhesion function.

Because the C-terminal PDZ-binding motif is intact in the Cys401Tyr mutant, its binding to PSD-95 appeared normal in the immunoprecipitation assay. On the contrary, the frameshift mutation is predicted to alter the ∼100 amino acids in the cytoplasmic part of the protein, including residues binding to cytoplasmic PSD-95 (figure 2B) and 14-3-3 proteins.^1,5,31^ Our binding assays showed that the frameshift mutation leads to a nonfunctional protein that is localized mainly in the ER and does not bind to LGI1 or to PSD-95.

The highly conserved exon 27 containing the frameshift deletion is subjected to alternative splicing (figure e-4), as are several other exons encoding parts of the cytoplasmic domain of ADAM22. Exon 27 is expressed in several regions of the human and mouse brain^13,26,27,32^ and has a progressive temporal expression pattern that differs from that of *ADAM22* exons that are not subjected to alternative splicing (figure e-5).^33^ Accounting for all exons, *ADAM22* expression is highest in postnatal stages (reference 1 and figure e-5). In embryonic stages, exon 27 is expressed in lower levels than other exons; the proportion of splice variants expressing exon 27 rapidly increases toward the end of the embryonic development and reaches a plateau at the first postnatal months. Exon 27 does not contain known peptide motifs, but its temporal expression pattern suggests that it contains residues playing a role in the CNS function in postnatal stages in particular. Reflecting the importance of LGI1–ADAM22/23 complexes in postnatal brain development, the *Lgi1*^−/−^, *Adam22*^−/−^, and *Adam23*^−/−^ mice start developing seizures around 10 postnatal days.^2,11–14^ The expression pattern of *ADAM22* and data from knockout mice are in line with the development of symptoms in our patient, who was apparently asymptomatic at birth, with the first symptoms appearing at approximately 2 months of age and the disease progressing rapidly thereafter.

Because not all *ADAM22* transcripts express exon 27, the frameshift mutation is likely not a total null mutation but rather a hypomorphic one, its effect increasing temporally. This may also explain why the compound heterozygous mutations in our patient were not lethal in early life, in contrast to *Lgi1*^*−/−*^, *Adam22*^*−/−*^, and *Adam23*^*−/−*^ mice, which die at 2–3 postnatal weeks.^2,11–14^ Furthermore, exon 27 is skipped from *ADAM22* in the PNS,^8,13,32^ which may explain why sural biopsy shows normally myelinated nerves in the proband, whereas the knockout mice present with marked hypomyelination of the peripheral nerves.^13^

One epileptogenic mechanism of LGI1–ADAM22 dysfunction could stem from the observations that loss of neuronal LGI1 reduces AMPAR-mediated synaptic transmission^1,2^ and that loss of ADAM22 reduces both AMPAR- and NMDAR-mediated currents in excitatory synapses.^5^ This role of the LGI1-ADAM22 complex as the regulator of functional maturation of excitatory synapses in postnatal stages is mediated by interaction between the PDZ-binding motif of ADAM22 and PSD-95.^5^ When the complex is dysfunctional, it leaves synapses immature^5^ and could promote seizure activity, as has been shown for a monoallelic LGI1 mutant.^4^ Reduction of synaptic AMPARs as a consequence of the disruption of LGI1-ADAM22 interaction has also been suggested^34^ to be the underlying epileptogenic pathomechanism in autoimmune limbic encephalitis, which manifests with amnesia and seizures and is caused by autoantibodies against LGI1.^35,36^ It remains unknown whether seizure activity contributes to the rapidly progressive degeneration of cortical regions in our patient or whether ADAM22 dysfunction has a direct negative effect on the development of neuronal connections, increasing seizure susceptibility.

Our data suggest that loss of ADAM22 function in the CNS underlies the severe rapidly progressing infantile-onset encephalopathy with epilepsy in our patient. Given that heterozygous *LGI1* mutations underlie ADLTE, previous studies have attempted to identify dominantly inherited mutations in *ADAM22* in ADLTE families.^37,38^ However, the fact that the parents of the proband—who are heterozygous carriers of loss-of-function mutations—do not have epilepsy indicates that heterozygous deleterious mutations are tolerated in *ADAM22* and that *ADAM22* is primarily a recessive disease gene. This hypothesis is supported by the presence of 9 truncating variants of the canonical *ADAM22* transcript in the ExAC database, whereas there are none for *LGI1*. Lack of rare biallelic mutations in *ADAM22* in other exome data sets (appendix e-1) indicates that *ADAM22*-associated encephalopathy is rare, which may partially be because complete knockout mutations of *ADAM22* are not tolerated and only hypomorphic mutations cause a phenotype similar to that in our study. Future studies are needed to confirm this genetic association and define the phenotypic presentations of ADAM22 dysfunction.

## Supplementary Material

Data Supplement

## GLOSSARY

ADLTE: autosomal dominant lateral temporal lobe epilepsy
cDNA: complementary DNA
CNV: copy number variant
ER: endoplasmic reticulum
ExAC: Exome Aggregation Consortium
GFP: green fluorescent protein
OFC: occipital frontal circumference
PNS: peripheral nervous system
