# Supplementary material for: Dysfunctional ADAM22 implicated in progressive encephalopathy with cortical atrophy and epilepsy
Source: Neurol Genet. 2016 Jan 21;2(1):e46. doi: 10.1212/NXG.0000000000000046 (PMC4817901; doi:10.1212/NXG.0000000000000046)
Supplement: Data Supplement [file supp_2_1_e46__index.html]

Data Supplement 

# Dysfunctional ADAM22 implicated in progressive encephalopathy with cortical atrophy and epilepsy

## Data Supplement

**Files in this Data Supplement:**

- Data Supplement - PDF
