# Supplementary material for: Dysfunctional ADAM22 implicated in progressive encephalopathy with cortical atrophy and epilepsy
Source: Neurol Genet. 2016 Jan 21;2(1):e46. doi: 10.1212/NXG.0000000000000046 (PMC4817901; doi:10.1212/NXG.0000000000000046)
Supplement: Data Supplement [file supp_2.1.e46_supplemental_data.pdf]

Supplemental Data

Dysfunctional ADAM22 implicated in progressive encephalopathy with cortical atrophy and epilepsy

Mikko Muona, MSc; Yuko Fukata, MD, PhD; Anna-Kaisa Anttonen, MD, PhD; Anni Laari, MSc; Aarno Palotie, MD, PhD; Helena Pihko, MD, PhD; Tuula Lönnqvist, MD, PhD; Leena Valanne, MD, PhD; Mirja Somer, MD, PhD; Masaki Fukata, MD, PhD; Anna-Elina Lehesjoki, MD, PhD

Contents

appendix e-1; table e-1; figures e-1, e-2, e-3, e-4, e-5; e-References e-1

Appendix e-1

Exome variant analysis under recessive and *de novo* inheritance models

Variants with the following Variant Effect Predictor[1] (Ensembl release 78) annotated variant consequences based on Sequence Ontology nomenclature were considered: missense variant, initiator codon variant, splice donor or acceptor variant, stop lost, stop gained, inframe insertion or deletion (inframe indels in tandem repeat regions[2] obtained from UCSC Genome Browser[3] were excluded), and frameshift variant. Variants within the transcripts included in the August 2014 version (release 17) of Consensus CDS (CCDS) project were considered. Deleteriousness of missense variants was estimated using CADD[4], PolyPhen-2 HumVar[5], SIFT[6] and MutationTaster[7]. Association of genes to Mendelian diseases was annotated based on the Online Mendelian Inheritance in Man, OMIM® database[8] and literature search.

In the recessive filtering (figure e-1), homozygous, compound heterozygous, or hemizygous variants with allele frequencies <1% in the three following variant databases were included: phase 3 release of the 1000 genomes project[9] (2535 individuals; http://www.1000genomes.org/), Exome Variant Server (EVS) NHLBI GO Exome Sequencing Project (version 0.0.25, 6503 individuals; http://evs.gs.washington.edu/EVS/), and Exome Aggregation Consortium (ExAC, v. 0.3, 60,706 individuals including over 3,200 Finnish exomes and a subset of the 1000 genomes and EVS data; http://exac.broadinstitute.org). Variants present as homozygous or hemizygous in any of the three variant databases were excluded.

Given that the healthy parents of the study subject were not exome sequenced, *de novo* mutations could not be assessed directly. Instead, in the exome variant filtering strategy aiming to identify pathogenic *de novo* variants, heterozygous variants absent

from the above three variant databases were included, after which the candidates were subjected to segregation analysis (figure e-1). Additionally, dbSNP build 138 (http://www.ncbi.nlm.nih.gov/SNP/) variants were excluded except those with clinical association in NCBI ClinVar (http://www.ncbi.nlm.nih.gov/clinvar/).

After applying the filtering criteria, low quality variants were excluded based on IGV visualization of sequence reads.[10] The qualifying variants were subjected to segregation analysis by capillary sequencing.

Even though mtDNA is not included in the SureSelect exome capture kit, an average of 35.7× sequencing coverage in the mitochondrial genome was obtained, due to the abundance of mitochondrial DNA in the cells. We called mtDNA variants using samtools.[11] The MITOMAP database (www.mitomap.org) was used to identify known mtDNA mutations and polymorphisms.

Cell surface binding assay

COS7 cells were seeded onto poly-*d*-lysine 12-mm cover slips in a six-well cell culture plate (3×10^5 cells/well) and co-transfected with LGI1-FLAG and ADAM22. At 24 h after transfection, cells were fixed with paraformaldehyde/120 mM sucrose/100 mM HEPES (pH 7.4) at room temperature for 10 min and blocked with PBS containing 10 mg/ml bovine serum albumin for 10 min on ice. The fixed cells were stained with anti-FLAG antibody, followed by Cy3-conjugated secondary antibody. Then, the cells were permeabilized with 0.1% Triton X-100 for 10 min, blocked with PBS containing 10 mg/ml BSA, and stained with anti-ADAM22 polyclonal antibody, followed by Alexa488-conjugated secondary antibody and staining with Hoechst dye (33342, Invitrogen). Fluorescent images were taken with a confocal laser microscopy system (Carl Zeiss LSM 510; Carl Zeiss). For double

staining of Ser799IlefsTer96 mutant and an ER marker (anti-KDEL antibody), transfected COS7 cells were fixed, permeabilized and blocked as described above. Then, cells were stained with anti-ADAM22 and anti-KDEL antibodies, followed by Alexa488- and Cy3-conjugated secondary antibodies, respectively.

Immunoprecipitation

The transfected HEK293T cells were washed with PBS and subsequently lysed with buffer A [20 mM Tris-HCl (pH 8.0), 1 mM EDTA, 1.3% Triton X-100 and 50 μg/ml PMSF]. The lysates were cleared by centrifugation at 10,000 *g* for 5 min at 4°C. The immune complexes were precipitated with FLAG-M2 agarose (Sigma-Aldrich) for 1 h, washed with buffer B [20 mM Tris-HCl (pH 8.0), 1 mM EDTA, 100 mM NaCl, 1% Triton X-100 and 50 μg/ml PMSF], and eluted with buffer B containing 0.25 mg/ml FLAG peptide[12]. The immunoprecipitates were separated by SDS-PAGE and gels were subjected to Western blotting.

Exome sequencing metrics

Exome sequencing of the proband produced 4.4 Gb of sequence with a mean coverage of 84.68× in the target regions, of which 96.2% and 92.8% were captured with at least 5× and 10×, respectively.

Lack of biallelic mutations in *ADAM22* in other datasets

To attempt to identify more patients with mutations in *ADAM22*, we utilized exome data from our 29 other in-house exomes from patients with severe epilepsy syndromes (unpublished data, Laari A., Muona, M. *et al.*). In addition, we accessed 178 exomes or whole genomes of epileptic encephalopathy cases generated in EuroEPINOMICS Rare Epilepsy Syndromes consortium and exomes from >1000 children included in the Deciphering Developmental Disorders project,[13] where 24%

of the cases present with seizures. However, we did not identify rare, biallelic mutations in *ADAM22* in these data.

Table e-1. Exome variants passing the filtering under the "*de novo*" and recessive hypotheses and subjected to segregation analysis.

Gene symbol	OMIM/inheritance	Position (GRCh37)	dbSNP ID	Ref/alt allele	Genotype	Segregation	ExAC quality filter	ExAC all frequency	ExAC Finns frequency	Consequence	Coding DNA change	Protein change	CADD, SIFT, PolyPhen, Mutation Taster[a]
**"*De novo*" hypothesis**													
SZT2	Epileptic encephalopathy early infantile 18 (615476)/recessive	1:43891807	-	C/T	Het	Inherited from unaffected parent	-	0	0	missense	c.3028C>T	p.Leu1010Phe	17.85,B,B,B
FMO1	-	1:171252283	-	G/T	Het	Inherited from unaffected parent	-	0	0	missense& splice_region	c.1184G>T	p.Gly395Val	26.9,D,D,D
WDR41	-	5:76749669	-	A/C	Het	Inherited from unaffected parent	-	0	0	missense	c.496T>G	p.Cys166Gly	24.5,B,D,D
ADAM22	-	7:87808344	-	AG/A	Het	See "Recessive hypothesis"	-	0	0	frameshift	c.2396delG	p.Ser799IlefsTer96	38,NA,NA,D
CDK5RAP2	Microcephaly 3 primary autosomal recessive, (604804)/recessive	9:123215986	-	A/C	Het	Inherited from unaffected parent	-	0	0	missense	c.2541T>G	p.Asp847Glu	7.077,B,PD,B
XPNPEP1	-	10:111651569	-	G/T	Het	Inherited from unaffected parent	Failed	2.48×10^{-5}	3.02×10^{-4}	missense	c.326C>A	p.Thr109Lys	32,D,D,D
GABRB3	Epileptic encephalopathy; {Epilepsy, childhood absence, susceptibility to 5}, (612269)/de novo	15:27018090	-	C/T	Het	Inherited from unaffected parent	Failed	0	0	missense	c.20G>A	p.Gly7Glu	17.72,B,B,D
SDK2	-	17:71386576	-	C/T	Het	Inherited from unaffected parent	Failed	8.40×10^{-5}	9.48×10^{-4}	missense	c.4042G>A	p.Ala1348Thr	25.5,B,D,D
**Recessive hypothesis**													
ADAM22	-	7:87765328	-	G/A	Het	Parents carriers for one *ADAM22* mutation each	Passed	1.66×10^{-5}	0	missense	c.1202G>A	p.Cys401Tyr	32,D,D,D
ADAM22		7:87808344	-	AG/A	Het		-	0	0	frameshift	c.2396delG	p.Ser799IlefsTer96	38,NA,NA,D
CPA4	-	7:129962500	rs200631467	G/T	Hom	Parents carriers, one unaffected sibling also homozygous for the variant	Passed	5.32×10^{-4}	3.02×10^{-3}	missense	c.1250G>T	p.Arg417Leu	10.71,B,B,B

Ref, reference; Alt, alternate; ExAC, Exome Aggregation Consortium; -, not available; Het, heterozygous; Hom, homozygous.
[a]A CADD score of >15 indicates deleteriousness for the variant. B, benign (not considered deleterious by the method); PD, possibly deleterious (applies to PolyPhen only); D, deleterious; NA, not available.
Mutation nomenclature is based on the following Ensembl/RefSeq transcripts: *SZT2*, ENST00000562955/NM_015284.3; *FMO1*, ENST00000354841/NM_001282692.1; *WDR41*, ENST0000296679/NM_018268.2; *ADAM22*, ENST00000265727/NM_021723.3; *CDK5RAP2*, ENST00000349780/NM_018249.5, *XPNPEP1*, ENST00000502935/NM_020383.3; *GABRB3*, ENST00000311550/NM_000814.5; *SDK2*, ENST00000392650/NM_001144952.1; *CPA4*, ENST00000222482/NM_016352.3.

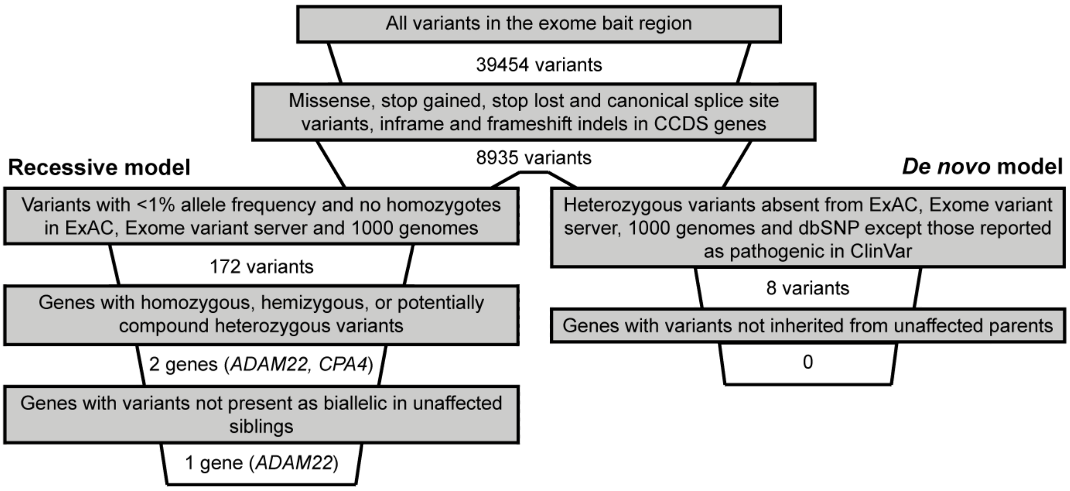

Figure e-1. Exome variant filtering under recessive and "*de novo*" models.

A flow chart showing the variant filtering process under recessive and "*de novo*" inheritance models. The number of variants or genes remaining after each step is presented. Variant or gene counts from the second last filtering step onwards in both recessive and "*de novo*" filtering have been counted after removing low quality variants based on visualization of sequence reads using Integrative Genomics Viewer (https://www.broadinstitute.org/igv/). The dramatic reduction in variant numbers in the allele population frequency based filtering is largely due to the high number of Finnish individuals in the ExAC database (>3,000) and the lower genetic heterogeneity in the genetically isolated Finnish population[14]. CCDS, Consensus Coding Sequence database; ExAC, Exome Aggregation Consortium.

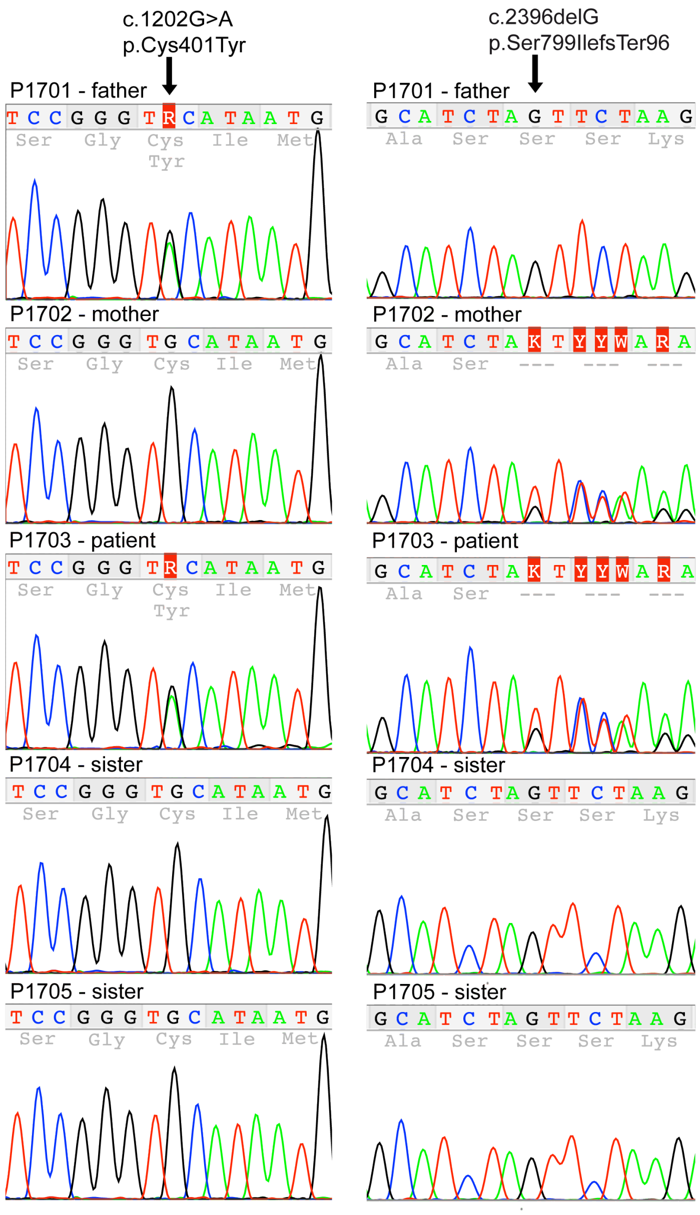

Figure e-2. Validation of *ADAM22* mutations.

Capillary sequencing chromatograms of *ADAM22* mutations c.1202G>A (left panel) and c.2396delG (right) in the patient and family members.

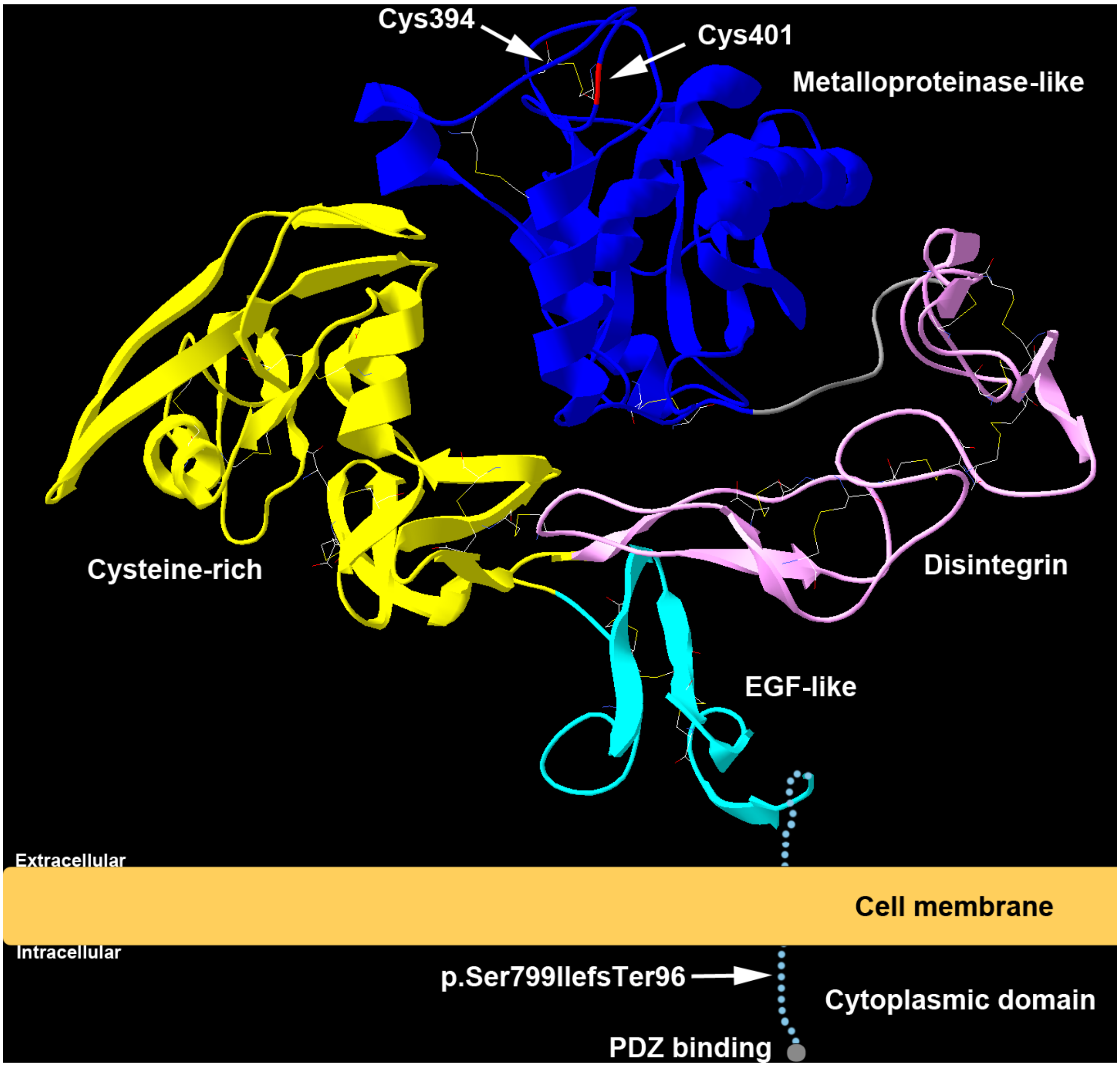

Figure e-3. Structure of the ectodomain of ADAM22 and the cysteine 401 residue.

Ribbon diagram showing the crystal structure of the four-leaf clover ectodomain of ADAM22 (based on Protein Data Bank submission 3G5C by Liu *et al.* [15]) combined with a cartoon (dotted line) of the transmembrane and cytoplasmic domains. The metalloproteinase-like, disintegrin, cysteine-rich and EGF-like domains are shown in blue, pink, yellow, and cyan, respectively. Arrow and red color in the protein model backbone indicate the Cys401 residue that is mutated in the patient. The Cys401 residue forms a disulfide bond with Cys394 in the wild-type ADAM22. Side chains of cysteine residues forming disulfide bonds are illustrated with thin stick models. Arrow in the cytoplasmic domain indicates the location of the frameshift mutation p.Ser799IlefsTer96 which abolishes the PDZ-binding motif (grey circle).

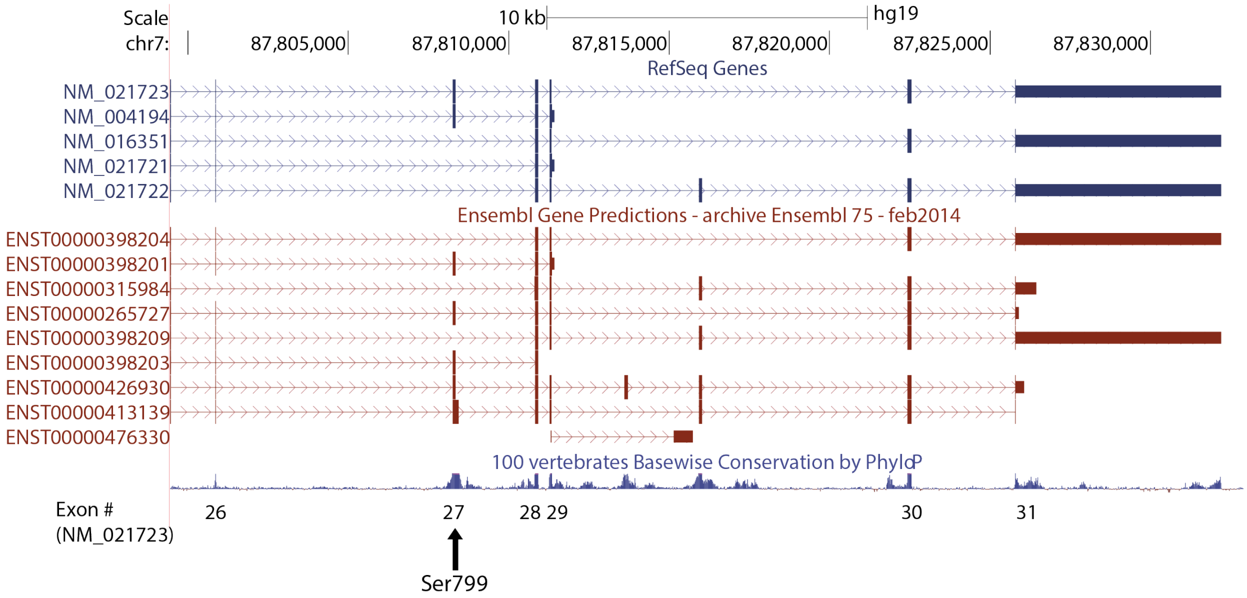

Figure e-4. Alternative splicing of *ADAM22* exons encoding cytoplasmic residues.

Representation of the exon structure of the cytoplasmic region of *ADAM22* in RefSeq and Ensembl databases demonstrating the alternative use of exons. Exons in RefSeq gene transcripts are illustrated with blue bars under "RefSeq Genes" and exons in Ensembl gene transcripts with red bars under "Ensembl Gene Predictions". Additional *ADAM22* splice variants in human and mice have been identified by others.[16,17] Numbers of exons included in the canonical RefSeq transcript (NM_021723) are shown in bottom. The arrow indicates exon 27, which contains the frameshift deletion (p.Ser799IlefsTer96). Basewise PhyloP conservation in 100 vertebrate species is presented in the blue graph under "100 vertebrates Basewise Conservation by PhyloP". Data is retrieved from the UCSC genome browser with hg19 (GRCh37) human reference genome (http://genome.ucsc.edu/cgi-bin/hgGateway).

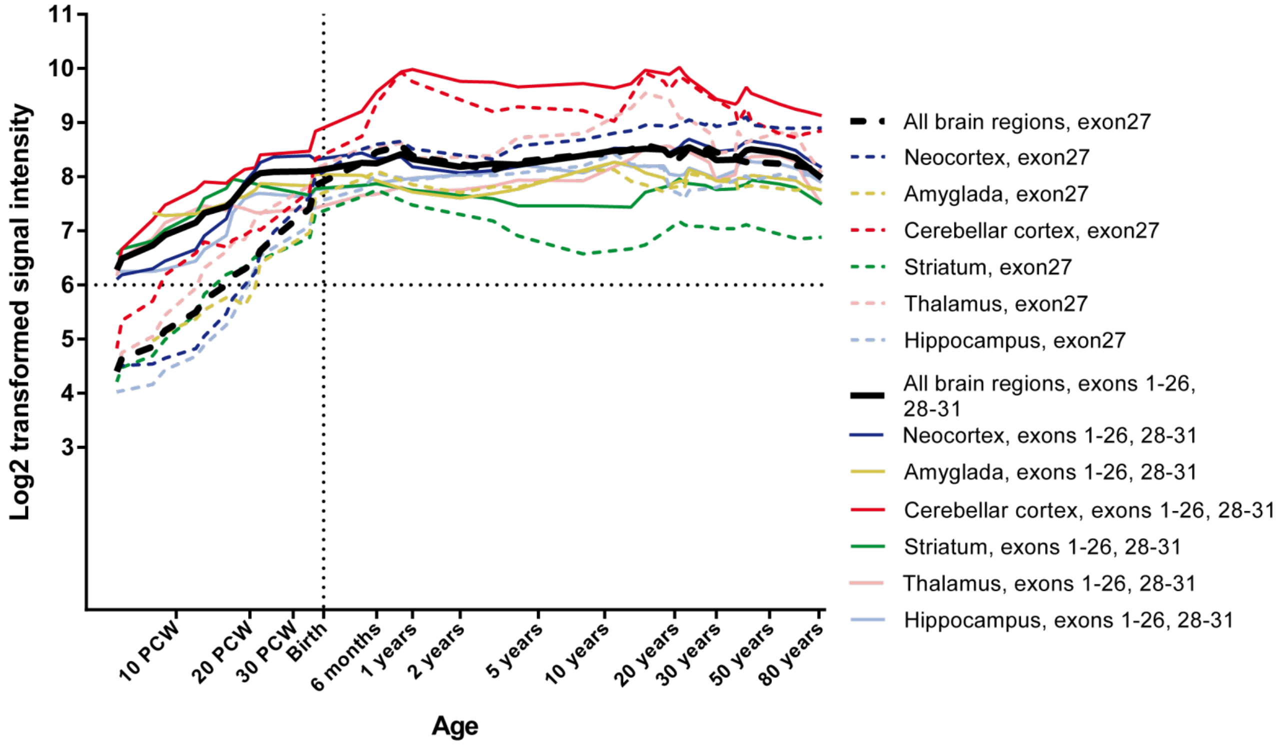

Figure e-5. Spatio-temporal expression of *ADAM22* exons in the human brain.

A graph presenting the spatio-temporal expression pattern of *ADAM22* exon 27 as well as the average of the expression of other *ADAM22* exons in the longest isoform (NM_021723). Smoothed dashed curves represent log_2-signal intensity of *ADAM22* exon 27. Smoothed solid curves represent the average of log_2-signal intensities of *ADAM22* exons 1-26 and 28-31. Color coding of brain regions is shown on the right. The horizontal dashed line at the log_2-transformed signal intensity value of 6 indicates the conservative threshold that was used by the authors to define an ‘expressed’ gene.[18] The graph was generated based on data released by the Human Brain Transcriptome project[18] using exon-level microarray data of the transcriptome in various brain regions (Gene Expression Omnibus accession ID GSE25219). The project used 1,340 tissue samples collected from 57 developing and adult post-mortem brains of clinically unremarkable donors. Similar results for *ADAM22* expression are obtained from BrainSpan database with exon array and RNA sequencing data from developing and adult human brain (BrainSpan: Atlas of the

Developing Human Brain, http://developinghumanbrain.org). PCW, postconceptual weeks.

e-References e-1

1. McLaren W, Pritchard B, Rios D, Chen Y, Flicek P, Cunningham F. Deriving the consequences of genomic variants with the Ensembl API and SNP Effect Predictor. Bioinformatics 2010;26:2069-2070.

2. Benson G. Tandem repeats finder: a program to analyze DNA sequences. Nucleic Acids Res 1999;27:573-580.

3. Kent WJ, Sugnet CW, Furey TS, et al. The Human Genome Browser at UCSC. Genome Res 2002;12:996-1006.

4. Kircher M, Witten DM, Jain P, O'Roak BJ, Cooper GM, Shendure J. A general framework for estimating the relative pathogenicity of human genetic variants. Nat Genet 2014;46:310-315.

5. Adzhubei IA, Schmidt S, Peshkin L, et al. A method and server for predicting damaging missense mutations. Nat Methods 2010;7:248-249.

6. Kumar P, Henikoff S, Ng PC. Predicting the effects of coding non-synonymous variants on protein function using the SIFT algorithm. Nat Protoc 2009;4:1073-1081.

7. Schwarz JM, Rodelsperger C, Schuelke M, Seelow D. MutationTaster evaluates disease-causing potential of sequence alterations. Nat Methods 2010;7:575-576.

8. Hamosh A, Scott AF, Amberger JS, Bocchini CA, McKusick VA. Online Mendelian Inheritance in Man (OMIM), a knowledgebase of human genes and genetic disorders. Nucleic Acids Res 2005;33:D514-D517.

9. The 1000 Genomes Consortium. An integrated map of genetic variation from 1,092 human genomes. Nature 2012;491:56-65.

10. Robinson JT, Thorvaldsdottir H, Winckler W, et al. Integrative genomics viewer. Nat Biotechnol 2011;29:24-26.

11. Li H, Handsaker B, Wysoker A, et al. The Sequence Alignment/Map format and SAMtools. Bioinformatics 2009;25:2078-2079.

12. Fukata Y, Lovero KL, Iwanaga T, et al. Disruption of LGI1–linked synaptic complex causes abnormal synaptic transmission and epilepsy. Proc Natl Acad Sci U S A 2010;107:3799-3804.

13. The Deciphering Developmental Disorders Study. Large-scale discovery of novel genetic causes of developmental disorders. Nature 2015;519:223-228.

14. Peltonen L, Jalanko A, Varilo T. Molecular genetics of the Finnish disease heritage. Hum Mol Genet 1999;8:1913-1923.

15. Liu H, Shim AHR, He X. Structural characterization of the ectodomain of a disintegrin and metalloproteinase-22 (ADAM22), a neural adhesion receptor instead of metalloproteinase: insights on ADAM function. J Biol Chem 2009;284:29077-29086.

16. Sagane K, Hayakawa K, Kai J, et al. Ataxia and peripheral nerve hypomyelination in ADAM22-deficient mice. BMC Neurosci 2005;6:33.

17. Gödde NJ, D'Abaco GM, Paradiso L, Novak U. Differential coding potential of ADAM22 mRNAs. Gene 2007;403:80-88.

18. Kang HJ, Kawasawa YI, Cheng F, et al. Spatio-temporal transcriptome of the human brain. Nature 2011;478:483-489.
